# De novo design of a pH-triggered self-assembled β-hairpin nanopeptide with the dual biological functions for antibacterial and entrapment

**DOI:** 10.1186/s12951-021-00927-z

**Published:** 2021-06-14

**Authors:** Qiuke Li, Jinze Li, Weikang Yu, Zhihua Wang, Jiawei Li, Xingjun Feng, Jiajun Wang, Anshan Shan

**Affiliations:** Laboratory of Molecular Nutrition and Immunity, The Institute of Animal Nutrition, Northeast Agricultural University, Harbin, People’s Republic of China

**Keywords:** Histidine functioned, pH-triggered self-assembled peptide, Antimicrobial activity, Entrapment property, β-hairpin structure

## Abstract

**Background:**

Acid-tolerant enteric pathogens can evade small intestinal acid barriers, colonize and infect the intestinal tract. However, broad-spectrum antibiotics are not the best therapeutic strategy because of the disruption of intestinal flora caused by its indiscriminate antimicrobial activity against beneficial and harmful bacteria. So that is what inspired us to combine pH regulation with nanotechnology to develop a pH-triggered site-targeted antimicrobial peptide with entrapping function.

**Results:**

A pH-triggered dual biological functional self-assembled peptide (SAP) was designed according to the features of amino-acid building blocks and the diagonal cation–π interaction principle. The results of characterization experiments showed that changes in pH conditions could trigger microstructural transformation of the nanopeptide from nanospheres to nanofibers. The subsequent antibacterial and toxicity experiments determined that SAP had great antimicrobial activity against *Escherichia coli*, *Salmonella typhimurium*, *Listeria monocytogenes*, and *Bacillus cereus* above 15.6 μg/mL under acidic conditions by disrupting bacterial membrane integrity, excellent biocompatibility in vitro even at 250 μg/mL and high tolerance in physical environment. Moreover, at peptide concentrations greater than 62.5 μg/mL, SAP showed the entrapment property, which played an important role in phagocytic clearance in infection forces. Meanwhile, the in vivo results revealed that SAP possessed excellent therapeutic effect and good biosafety.

**Conclusions:**

Our study revealed the antibacterial activity of a short β-hairpin forming self-assembled peptide, and established an innovative design strategy for peptide-based nanomaterials and a new treatment strategy for gastrointestinal bacterial infections.

**Graphic Abstract:**

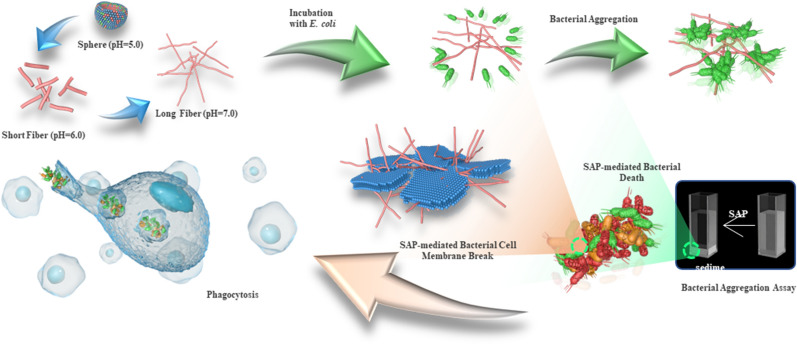

**Supplementary Information:**

The online version contains supplementary material available at 10.1186/s12951-021-00927-z.

## Background

Acid tolerance of enteric pathogens such as *Escherichia coli* and *Salmonella* plays an important role in evading the acid barrier of the stomach (pH 1.5–2.5) and small intestine (pH 4.0–6.0), leading to bacterial colonization and infection in the intestinal tract, which results in bacterial gastroenteritis and causes a range of symptoms, including vomiting, severe abdominal cramps and diarrhea [1]. However, it is widely accepted that antibiotic therapy is not the best therapeutic strategy for enteric infection due to the increased drug- resistance of bacteria and disruption of microbial communities as a consequence of broad-spectrum therapy [2, 3]. Inspired by the above, an acid-triggered antibacterial treatment strategy might be developed to target specific acid resistance to reduce side effects and avoid secondary infections [4].

Over the past decade, functionalized self-assembled nanomaterials have been constructed for diverse potential applications with the advancement of nanotechnology [5]. Among these molecules, peptide building blocks are widely evaluated in nanomedicine, such as disease diagnostics, drug delivery [6, 7], tissue scaffolds [8, 9] and antimicrobial agents [10, 11] due to their inherent biocompatibility, resistance to digestion and goal-oriented operability. This operability is derived from the controllability of structural features by molecular chemistry, the assembly environments (pH [12], enzymes [13], solvents [14], coassembling molecules [15], and temperature), and assembly kinetics, which could be utilized to fulfill various demands in medical applications [5]. In particular, pH-responsive self-assembly is a unique strategy, superior to those requiring external stimuli, which is auto-triggered by a change in microenvironment pH and achieves site-targeting identification via the programmed response of nanoparticles [7]. Microenvironment pH-triggered deprotonation-protonation events play a decisive role in the structural and functional features of peptide nanoparticles, which show the high potential of this stimuli system for the development of nanomedical drugs, since this site-targeted strategy would improve the treatment efficiency in particular cases, where a deviation in the pH values from neutral is observed [16, 17]. Therefore, it is necessary to combine pH-switching strategies with nanotechnology to target specific acid resistance mechanisms in the case of acid-tolerant enteric pathogenic infections.

To date, the self-assembling peptide design strategy can be summarized into four main categories: peptide amphiphiles (PAs), aromatic short peptide derivatives, α-helices, and β-sheet peptides [18]. Each of them has been developed for various biofunctionalized nanoparticles based on their molecular amphiphilicity, structural compatibility, complementarity and environmental responsiveness [19]. Among them, β-sheet peptides are more likely to self-assemble into fibrous structures due to the massive presence of hydrogen bonds [5], as is seen in amyloid diseases such as Alzheimer’s disease and Parkinson’s disease [20]. These β-fibrous proteins have been determined to show potent antimicrobial activity and propensity for entrapping and agglutinating bacteria [21], preventing bacterial invasion into histocytes and promoting pathogen phagocytosis [22], which play essential roles in human innate immunity to maintain microbiota homeostasis of the intestine [23, 24]. However, few studies have reported the pH-triggered microstructural transformation and biological properties of β-hairpin self-assembled peptides.

**Scheme 1:**
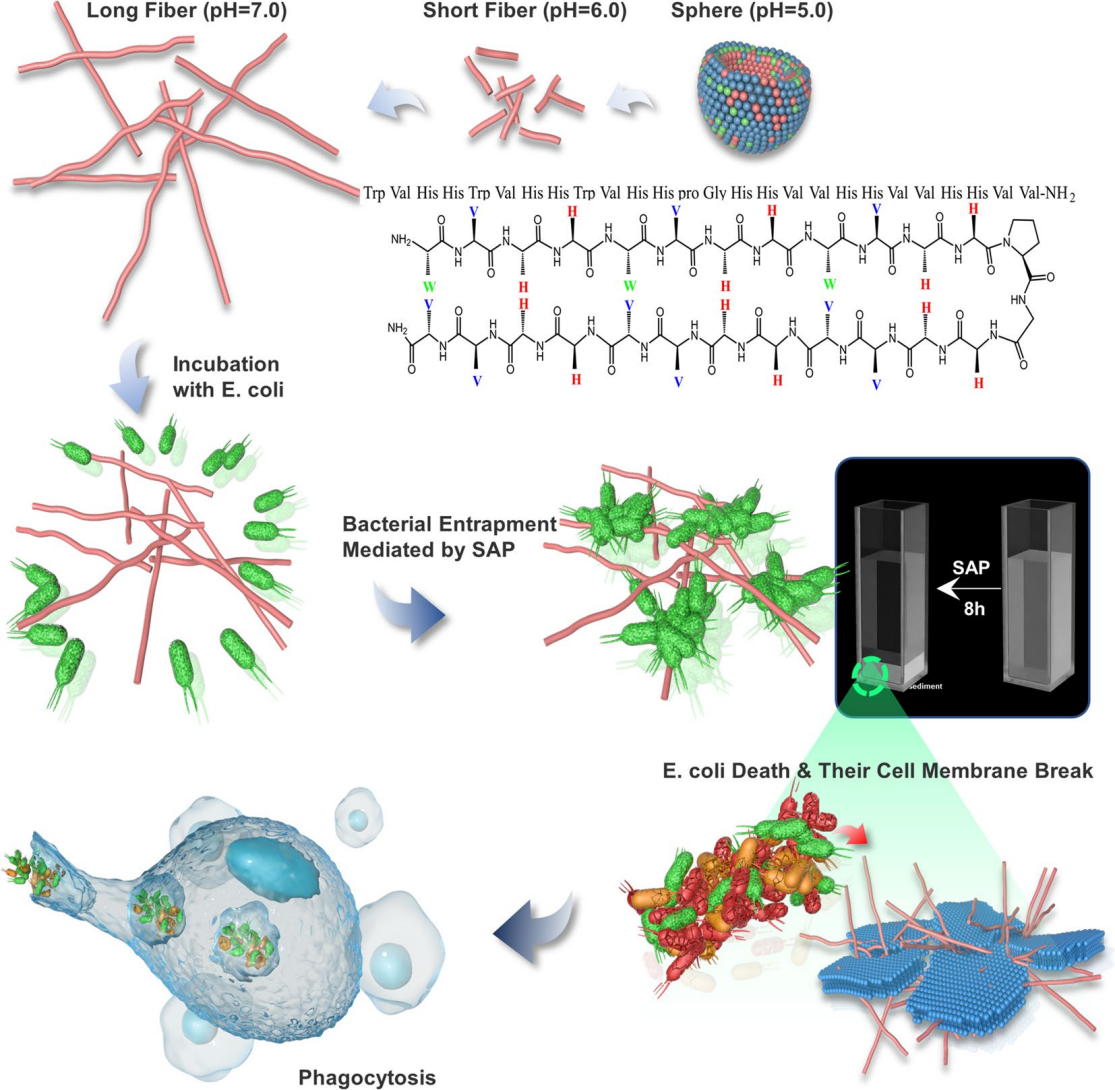
Schematic illustration of the pH-triggered microstructural transformation of the β-sheet self-assembled peptide and its biological functions

Hence, we adopted a positional relation approach to de novo design a β-hairpin pH-triggered self-assembled peptide (SAP). The self-assembling capability and pH-triggered transformation of nanostructures were first presented. Antimicrobial assays, agglutination activity tests and cytotoxicity tests were performed to assess the biological functions of SAP and correlate potential interactions between biological activity and microstructure with changes in the pH environment. Subsequently, toxicity and therapeutic activities in vivo were assessed to explore the application potential of SAP. This study designed a novel β-hairpin pH-triggered self-assembled peptide (SAP) to achieve the aim of switching its biological activity ‘on’ and ‘off’ in response to changes in pH, which showed the pH-switchable structural transformation and biological activities, and exhibited effective in vivo therapeutic effect, paving the way for further development of peptide-based supramolecular nanoparticles in nanomedicine (Scheme 1).

## Results and discussion

### Design principles and molecular basis of SAP

**Fig. 1:**
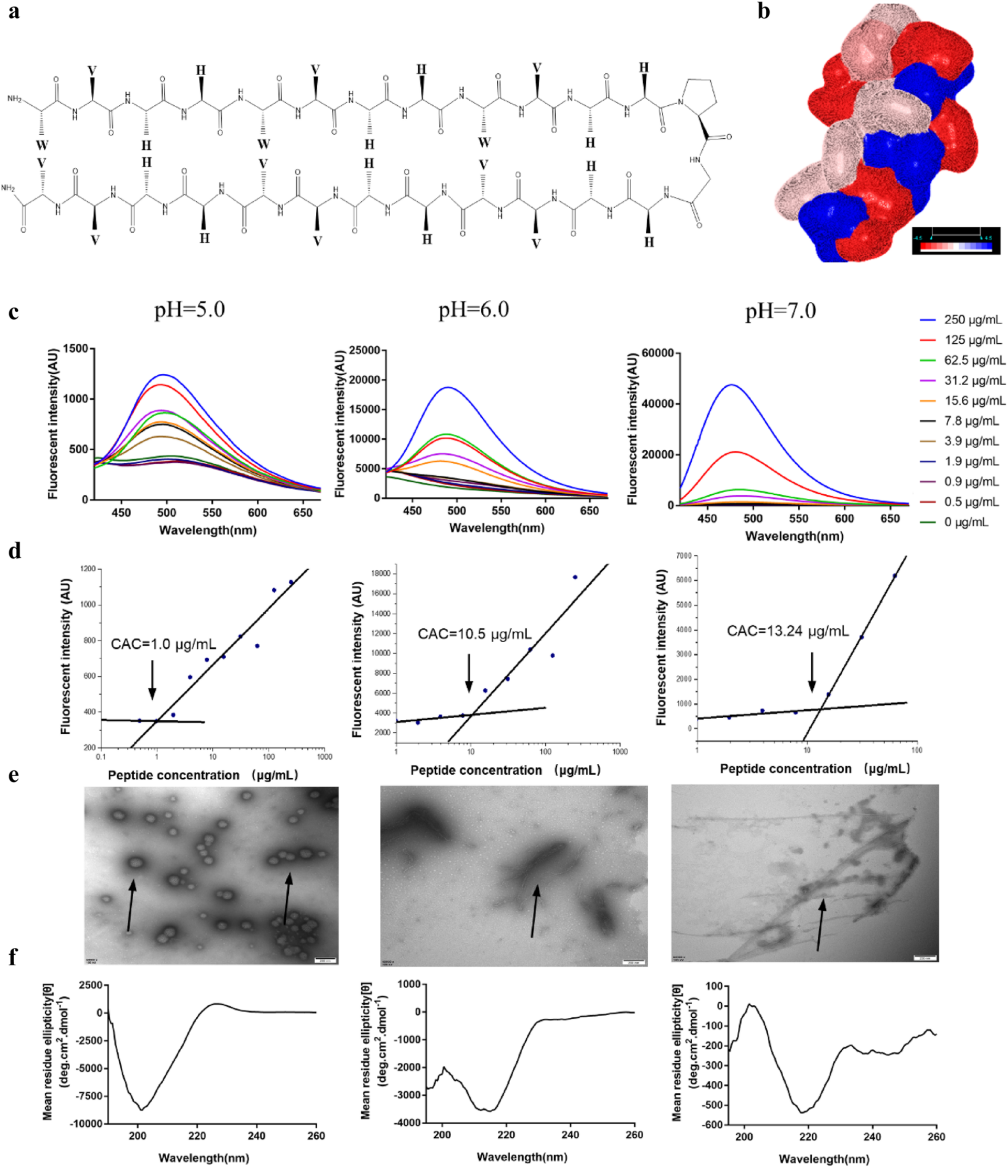
**a** Schematic of the SAP. **b** The three-dimensional structure of SAP modeling by http://zhanglab.ccmb.med.umich.edu/I-TASSER. The hydrophobicity spectrum: red-white-blue (− 4.5 ~  + 4.5). **c** Concentration-dependent self-assembly of the SAP. ANS fluorescence binding assay for the SAP at different concentrations at pH 5.0, 6.0 and 7.0. **d** The critical aggregation concentrations of the SAP at pH 5.0, 6.0 and 7.0. **e** TEM images and **f** CD spectra of SAP nanoparticles under three pH conditions

The SAP was established by considering specific amino acids with special properties and the diagonal cation-π interaction. SAP contained aliphatic or aromatic amino acids (9 Val and 3 Trp) and basic amino acids (12 His) as a pH switch providing multiple interaction capabilities at different pH values in the supramolecular self-assembly system. It was reported that His contains an aromatic imidazole side chain, which provides cation–π (when His is protonated, His^+^), π–π stacking, hydrogen–π and hydrogen bonding with other molecules or coordination interactions with metallic cations to play a significant role in protein–protein interfaces and catalysis [25]. Under the acidic condition, the side chains were protonated, which increased the positive charges of the SAP molecules, prompting them to attract with the anionic bacterial membrane. Meanwhile, His, as a pH switch, changed the hydrogen bond energy of self-assembly system when it was protonated and triggered the microstructural transformation of self-assembled peptide. Under the neutral condition, the imidazole ring deprotonated and could be used as a hydrogen donor and acceptor, assisting SAP molecule aggregation into supramolecular structures. In this study, acidic (pH = 5.0 and pH = 6.0) and neutral (pH = 7.0) conditions were selected as model environments for investigating the transformation of nanostructures and biological functions. Besides, Trp with an aromatic ring, which is reported to serve as hydrogen bond donors and acceptors, facilitates the formation of hydrogen bonds [19], provides hydrophobicity strength for assisting with the formation and stability of nanostructures via π–π stacking and plays an important role in acceleration and stabilization of amyloidogenic assemblies [10]. Meanwhile, Trp-rich peptides act on the bacterial cytoplasmic membrane [26], interact strongly with hydrophobic phospholipid tails, and facilitate peptide insertion into the phospholipid bilayer for permeabilization or pore formation [27]. The hydrophobic amino acid Val was selected because of its strong propensity for forming intermolecular hydrogen bonds, as Val typically possessing high β-sheet forming propensities, which easily leads to the formation of fibers [28]. Furthermore, Trp and His were adapted in two consecutive non-H-bonded sites participating in a diagonal cation–π interaction, which provided driving force to maintain and stabilize the β-hairpin structure [29, 30]. Then, equal amounts of His and Val were distributed to other sites to possess enough positive and hydrophobic properties for antimicrobial activity. Additionally, one of the type II’ β-turns, ^D^Pro–Gly, was selected as a linker to connect two strands to form a β-sheet structure. The C-terminus of the peptides was aminated to further enhance stabilization. The sequence of SAP and the three-dimensional structure modeling were shown in Fig. 1a, b.

### pH-triggered microstructural transformation of SAP

The self-assembling ability and the critical aggregation concentration (CAC) were investigated via a 1-anilino-8-naphthalene sulfonate (ANS) fluorescence assay, because the fluorescent intensity is enhanced when binding to macromolecules. The CAC was determined as described by Fung et al. the ANS fluorescence intensity values at 475 nm were selected, the profile with different peptide concentrations was fitted with two straight lines, and the peptide concentration of intersection indicated the CAC [19]. The results were shown in Fig. 1c. The ANS fluorescence intensity increased with increasing peptide concentration and aggregation strength at pH 5.0, 6.0 and 7.0, indicating that the SAP molecule could aggregate to form a supramolecular organization under all three pH environments. The CACs of the SAP at different pH environments were shown in Fig. 1d. SAP was able to assemble above the concentrations of 1.0 μg/mL,10.5 μg/mL and 13.24 μg/mL at pH 5.0, 6.0 and 7.0, respectively, demonstrating that the lower the pH was, the better the aggregation ability of SAP would be.

The microstructure of SAPs in different environments was investigated via transmission electron microscopy (TEM). The TEM results showed that SAP at a concentration of 15.6 μg/mL self-assembled into spherical micelles at pH 5.0 (Fig. 1e). Spherical micelles transformed into nanofibers after adjusting the pH to 6.0 and 7.0. Compared with the neutral condition, the nanofibers were shorter at pH 6.0, which indicated that the microstructure of the SAP gradually transformed from spheres to nanofibers with the change in pH conditions, and as the pH increased, the microstructure of the SAP transformed into longer nanofibers. Then, the secondary structure transition of the SAP was investigated using circular dichroism (CD) spectroscopy under different pH conditions. As Fig. 1f shown, the spectrum of the SAP at pH 5.0 had a negative band at 200 nm indicating that the peptide adopted a random coil configuration [31], while increasing the pH, it showed a positive peak at 199 nm and a strong negative peak at 218 nm, indicating that the peptide adopted a large amount of β-sheet structure due to the increase in intramolecular and intermolecular hydrogen bonding among the peptide [32]. These data were in direct agreement with the transition of the microstructure when changing the pH environment. Below the acidic condition (pH = 5.0), the side chains of His were protonated and positively charged aromatic His residues changed hydrogen bonding energies between the residues and prevented the formation of rigid β-sheets. Thus, the SAP adopted a random coil configuration. The stronger hydrophobic interactions provided by Trp and Val promoted the SAP to form micelles of finite size, which followed a closed association pathway above a critical micellization concentration [33]. With the increase in the pH (from acidic to neutral pH), the imidazole ring was protonated, which could be either in the neutral or positively charged form, involving in the conformation of hydrogen bonding interactions as hydrogen bond donors and acceptors [34]. Increasing the hydrogen bonding energy promoted the SAP to follow an open association scenario, resulting from the step-by-step aggregation of molecules to form board distribution of β-sheets. The bilayer was formatted with the hydrophobic faces of two layers, which buried onto each other to separate the hydrophobic moieties from water [35]. And with the formation of hydrogen bonding interactions between adjoining hairpins, fibril structures of the SAP were formed and extended the length of fibers with the increase of pH [36].

A thioflavin T (ThT) fluorescence assay also confirmed this conformational transformation of SAP. The fluorescence values of the peptide were the lowest at pH 5.0 and increased with increasing pH, indicating that the peptide gradually formed an amyloid protein fiber structure with increasing pH (Additional file 1: Figure S3a). The hydrodynamic sizes of the SAP micelles at pH 5.0 were evaluated by dynamic light scattering (DLS) using a Zetasizer Nano ZS90, which presented hydrodynamic diameter of ~ 119 nm (Additional file 1: Figure S3b). The measured ζ-potential of the SAP nanoparticles decreased from + 26.2 to + 1.83 mV upon an increase in the pH from 5.0 to 7.0, which indicated that SAP fibers tended to transform to a neutral status (Additional file 1: Figure S3c).

### Antimicrobial activity, antimicrobial mechanism and in vitro toxicity of SAP

**Fig. 2:**
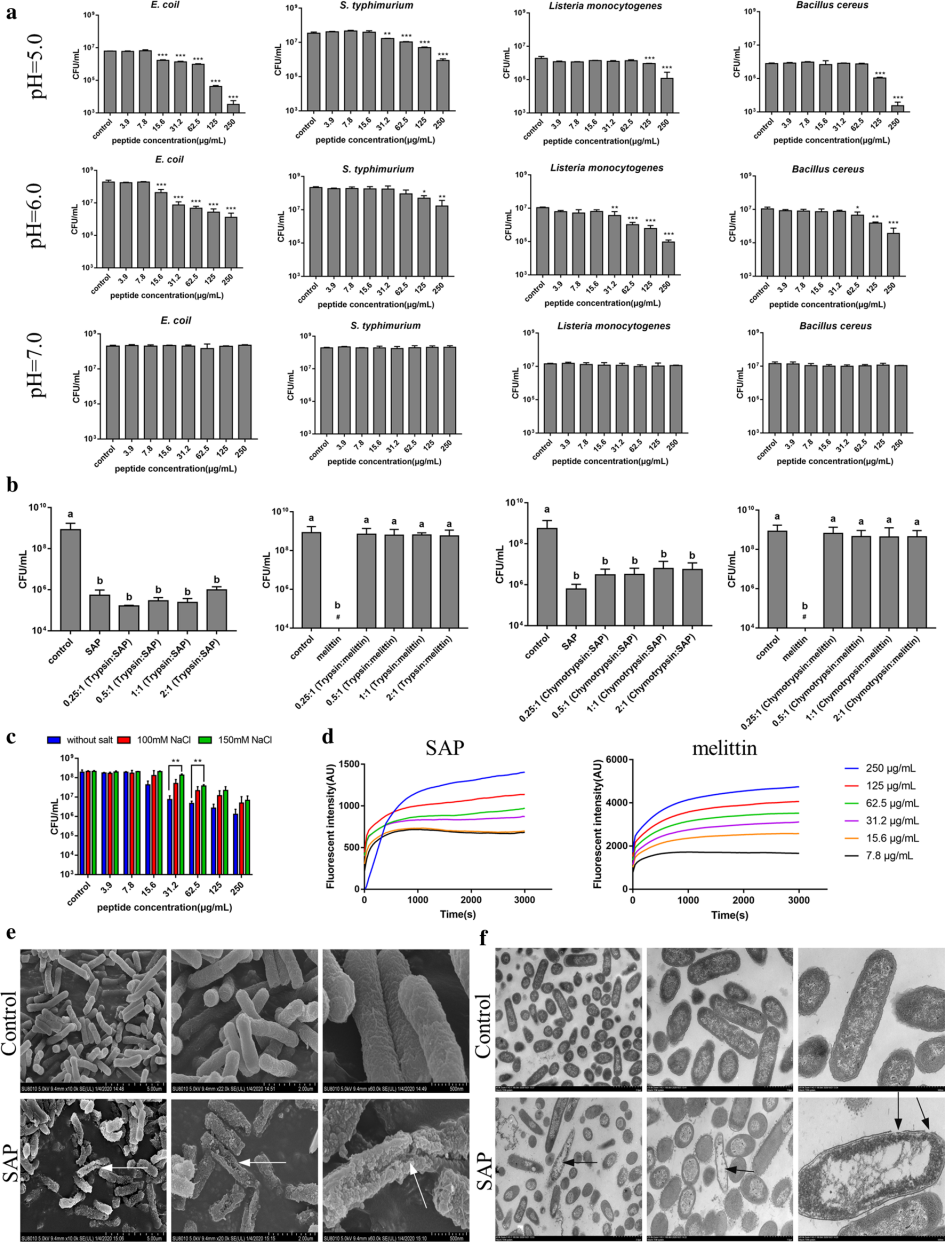
**a** Antibacterial activity of the SAP against *E. coli* ATCC 25922, *S. typhimurium* ATCC 14028, *Listeria monocytogenes* CGMCC 1.10753 and *Bacillus cereus* CGMCC 1.932 in three pH environments (mean ± SD, n = 3). Compared to control: * *p* < 0.05, ** *p* < 0.01, *** *p* < 0.001. **b** Antibacterial activity of the SAP and melittin at pH 6.0 against *E. coli* ATCC 25922 after incubation with trypsin and chymotrypsin at mass ratios of 2:1, 1:1, 0.5:1 and 0.25:1 (enzyme: peptide), respectively for 1 h at 37 °C (mean ± SD, n = 3). # indicated no colony formation. **c** Antibacterial activity of the SAP against *E. coli* ATCC 25922 in the presence of 100 mM and 150 mM NaCl at pH 6.0 (mean ± SD, n = 3) Compared to the control: ** *p* < 0.01. **d** Cytoplasmic membrane potential variations of *E. coli* ATCC 25922 treated by SAP and melittin at different concentrations. **e** SEM micrographs of *E. coli* ATCC 25922 treated with 250 μg/mL SAP for 30 min at pH 6.0 or not. **f** TEM micrographs of *E. coli* ATCC 25922 treated with 250 μg/mL SAP for 30 min at pH 6.0 or not

The antimicrobial activity of the self-assembled peptide was tested by colony counting experiments against clinically prevalent bacterial species, including *Escherichia coli*, *Salmonella typhimurium*, *Listeria monocytogenes,* and *Bacillus cereus.* After treatment of bacterial cells [(2–5) × 10^5^ CFU/mL] with the peptide at different concentrations ranging from 3.9 μg/mL to 250 μg/mL for 3 h, 50 μL of the bacterial solution was further continuously diluted, and a 10 μL aliquot from each dilution was plated on TSB agar plates. As shown in Fig. 2a, the SAP displayed remarkable antimicrobial activities under acidic conditions above the CAC. Especially for the representative strain *E. coli* ATCC 25922, the SAP at the highest concentration tested (250 μg/mL) exhibited highly effective activity, which reduced survival by approximately three orders of magnitude at pH 5.0 and two orders of magnitude at pH 6.0. The same results were observed in antibacterial experiments against *S. typhimurium* ATCC 14028, in which 250 μg/mL SAP reduced the survival by approximately two orders of magnitude and one order of magnitude at pH 5.0 and 6.0, respectively. For gram-positive bacteria *Listeria monocytogenes* CGMCC 1.10753, 250 μg/mL SAP reduced two orders of magnitude at pH 5.0 and 6.0. For *Bacillus cereus* CGMCC 1.932, 250 μg/mL SAP reduced one and two orders of magnitude at pH 5.0 and 6.0, respectively. On the contrary, no detectable antibacterial activity against bacteria was exhibited at various concentrations at pH 7.4 (Fig. 2a). The above results showed that the formation of nanostructure was indispensable for SAP to inhibit the bacterial proliferation, and the lower the pH was, the better the antibacterial effect of SAP would be, indicating that spherical micelles could effectively inhibit the bacterial growth and that the antibacterial effect decreased gradually with the transformation from spherical nanoparticles to fibers. The shape of the nanostructure influences the antimicrobial properties, and spherical nanoparticles are more effective than fiber nanoparticles [28]. Additionally, the net charge of the nanoparticle might contribute to the altered antibacterial activity. The electrostatic interaction between cationic AMPs and anionic bacterial membranes is considered important to the driving force for bacterial-peptide adsorption [37]. Under acidic conditions, His was protonated, which enhanced the positive electricity of the nanoparticle, and increased the electrostatic adsorption of peptides and the bacterial surface [38]. Moreover, we discovered an interesting phenomenon in the course of the antibacterial experiment that bacterial clusters could be observed after treatment with SAP, which indicated that SAP could entrap bacteria and promote bacterial agglutination, and we would explore additional experiments to investigate this interesting finding, subsequently.

In addition, self-assembled molecules have been reported to overcome proteolytic degradation challenges. To evaluate the properties of SAP, peptides were incubated with trypsin and chymotrypsin at mass ratios of 2:1, 1:1, 0.5:1 and 0.25:1 (enzyme: peptide) for 1 h at 37 °C and then the antimicrobial activity of the hydrolyzed peptide was assessed. The results were shown in Fig. 2b, SAP maintained its antibacterial activity after incubation with trypsin compared with unhydrolyzed peptide, even when the mass ratio of enzyme to peptide was 2:1 (*p* > 0.05), suggesting that SAP could resist the hydrolysis of trypsin. This is because trypsin preferentially cleaves at basic residues (Arg and Lys) rather than His [38]. And there were no significant differences, despite the slight increase in the number of colonies of *E. coli* after treatment with the hydrolyzed peptide compared with after treatment with the unhydrolyzed peptide (*p* > 0.05), indicating that nanoparticles were slightly hydrolyzed by chymotrypsin in a concentration-dependent manner. This may be due to Trp contained in the SAP, which is prone to digestion by chymotrypsin [39]. However, melittin, which was used as a control, was completely hydrolyzed by trypsin and chymotrypsin after incubation for 1 h and became inactive against *E. coli* even at the maximum tested concentration (250 μg/mL). This is because melittin contains Leu, Trp, Lys and Arg, which are prone to be hydrolyzed by chymotrypsin and trypsin (Fig. 2b).

To assess the salt sensibility of the SAP *in vitro*, the antibacterial activity in the presence of NaCl was monitored using a viable colony count assay against *E. coli* ATCC 25922 as the model strain. As shown in Fig. 2c, there was a slight increase in the number of colonies of *E. coli* after treatment in the presence of NaCl, but no significant differences were detected in the treatment of SAP above 15.6 μg/mL in the presence of 100 mM NaCl and above 125 μg/mL in the presence of 150 mM NaCl, compared with the control in the absence of NaCl, suggesting that the presence of NaCl could impact the biological function of the nanoparticles. This may be due to the effects of the electrostatic interaction between antimicrobial peptides and bacterial membranes in the presence of Na^+^ [40]. Moreover, NaCl also showed a protective effect on cell survival at pH 5.0 and there was no significant difference after treatment in the presence or absence of NaCl above 31.2 μg/mL (except 250 μg/mL) (Additional file 1: Figure S4) [37].

AMPs are considered less likely to develop drug resistance because they could compromise the integrity of microbial cell membranes, thereby evading the pathways by which bacteria develop rapid antibiotic resistance [41]. Based on the above results, *E. coli* was chosen as tested strain because SAP showed the best antibacterial activity against *E. coli* than other bacteria, and pH 6.0 was selected as the model condition to investigate the antibacterial mechanism because under this pH, the SAP showed antibacterial activity and the *E. coli* showed substantial growth improvement. Membrane depolarization and permeation were evaluated using a 3,3′-dipropylthiadicarbocyanine iodide (diSC3-5) fluorescence assay. DiSC_3-5_, a membrane potential-sensitive dye, displays increased fluorescence upon changing the cytoplasmic membrane potential. Figure 2d showed that rapid dose- and time-dependent increases in the fluorescence intensity induced by various concentrations of SAP as well as melittin, a typical membrane disruption peptide. Collectively, these results demonstrated that SAP could lead to membrane potential perturbation and pore formation. Scanning electron microscopy (SEM) and TEM were used to evaluate the effect of SAP treatment on bacterial morphology and intracellular ultrastructural alterations at the highest test concentration, respectively. As shown in Fig. 2e, in comparison with the control, the *E. coli* membrane induced by treated with 250 μg/mL SAP at pH 6.0 was seriously damaged, while SAP-untreated *E. coli* showed a complete membrane surface. Numerous nicks and tears were evident in the cell membrane of the 250 μg/mL SAP-treated *E. coli*, where membrane disintegration and clumping were distinctly observed and debris and lysis of the cells were also evident. For intracellular ultrastructural alterations of *E. coli* after treatment with SAP, Fig. 2f showed the notable separation of the cytoplasmic membrane and outer membrane, disruption of the outer membrane and the formation of obvious cavities within the *E. coli*, which determined that bacteria lost the intracellular content via the pores after treatment with nanopeptides in an acidic environment. In contrast, enriched cellular content and complete outer and inner membranes were observed in *E. coli* without nanopeptide treatment. According to the SEM, TEM and DiSC_3–5_ results, the distinct morphological disruption caused by SAP illustrated that the bacterial membrane was an important target of the antibacterial activity of nanopeptides in an acidic environment.

**Fig. 3:**
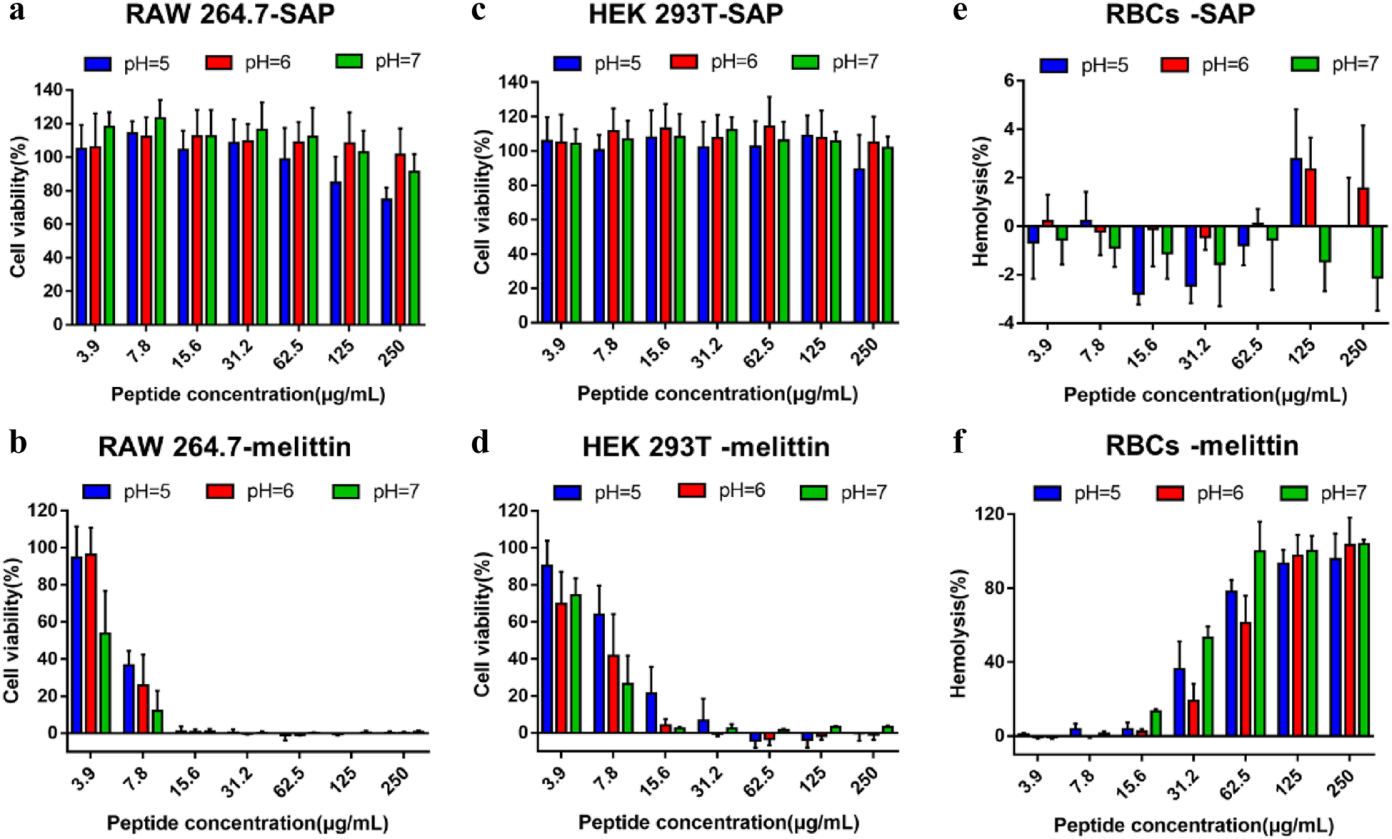
Cytotoxicity of various concentrations of the SAP and melittin against RAW264.7 cells, HEK293T cells and RBCs under three pH conditions (mean ± SD, n = 3)

In addition, the biocompatibility assessments of peptide are the prerequisite for further application. Thus, for testing the application potential of SAP, RAW264.7 cells, HEK293T cells and mouse red blood cells were selected as model cells for evaluating the safety of SAP at pH 5.0. 6.0 and 7.0. The cytotoxicity of SAP against RAW264.7 and HEK293T cells was tested via a 3-(4,5-dimethyl-2-thiazolyl)-2,5-diphenyl-2-H-tetrazolium bromide (MTT) dye reduction assay. For RAW264.7 macrophage cells, the results showed that the cell survival rate was maintained above 80% after treatment with SAP at pH 6.0 and 7.0, even at the highest test concentration of 250 μg/mL, but at pH 5.0, the cell survival rate showed a concentration-dependent reduction, which was approximately 70% after treatment with 250 μg/mL SAP for 4 h (Fig. 3a)**.** This result was sharply in contrast to the result obtained for melittin, in which the cell survival rate decreased to 30%, 25% and 10% after treatment with 7.8 μg/mL melittin at pH 5.0, 6.0 and 7.0, respectively (Fig. 3b). Similar to RAW264.7 cells, the survival rates of HEK293T cells induced by SAP were above 80% (Fig. 3c), but the cell survival rates induced by 7.8 μg/mL melittin decreased to 60%, 40% and 25% at pH 5.0, 6.0 and pH 7.0, respectively (Fig. 3d).

In the hemolysis assay, mouse erythrocytes were added into different concentrations of the peptide in three environments and the toxicity was determined by the amount of hemoglobin released by the lysis of erythrocytes. The results were shown in Fig. 3e, the SAP induced no hemolysis against mouse red blood cells at pH 5.0, 6.0 and pH 7.0, not exceeding 5% even at highest concentration 250 μg/mL, but 62.5 μg/mL melittin caused hemolysis exceeding 50% at pH 5.0, 6.0 and 7.0, respectively (Fig. 3f).

Based on the results of cytotoxicity and hemolysis assay, spherical nanoparticles showed higher toxicity on cells than nanofibers, indicating that the transition of peptides to fibers reduces the interaction with the membrane, which was validated by the antimicrobial activity results. Moreover, compared to the natural peptide melittin, the SAP showed high biocompatibility and fewer side effects, which indicated that the SAP has the potential for clinical use as a promising therapeutic medicine.

### Bacterial agglutination and phagocytosis mediated by SAP

**Fig. 4:**
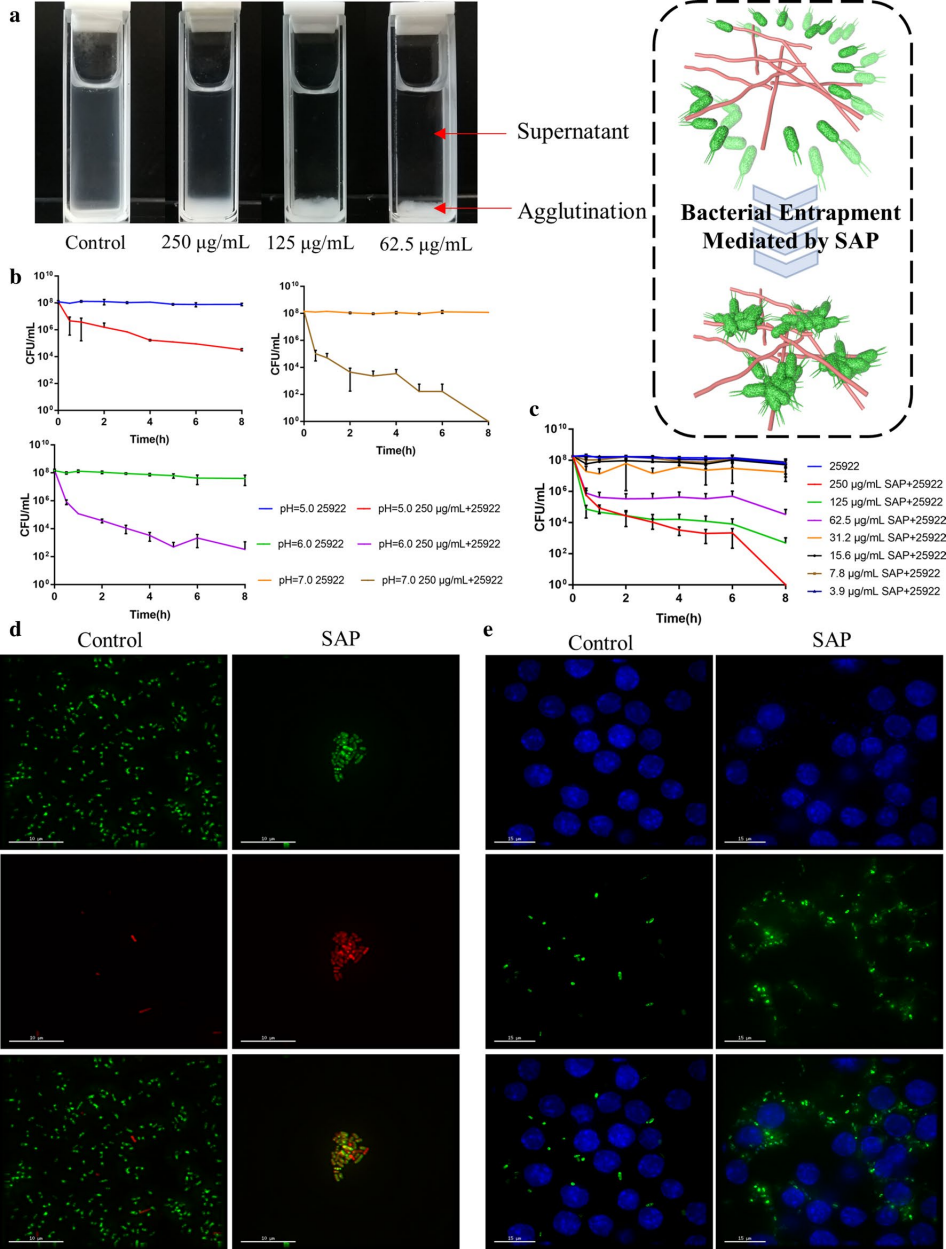
**a** The entrapment phenomenon caused by SAP. **b** Plots of colony forming units (CFU/mL) of the supernatant of *E. coli* ATCC 25,922 treated with 250 μg/mL SAP for 8 h in HEPES buffer at pH = 5.0, 6.0 and 7.0. **c** Plots of colony forming units (CFU/mL) of bacterial supernatant treated with different concentrations of SAP for 8 h in HEPES buffer at pH = 6.0. **d** Deltavision OMX SR fluorescence microscopic images analysis of *E. coli* strain BL21 consisting of PET-28a-EGFP plasmid treated with 250 μg/mL SAP or not. **e** Fluorescence microscopic images analysis of phagocytosis of *E. coli* (green) particles pretreated with 250 μg/mL SAP for 1 h by RAW 264.7 cells (DAPI, blue) or not

Based on the phenomenon that SAP could promote bacterial agglutination in the antibacterial assay, bacterial agglutination assay was performed to assess the entrapment function (Fig. 4a), which was evaluated by the turbidity of the bacterial solution and the number of colonies forming units of bacterial supernatant after SAP treatment as Phoom et al. described [24]. To investigate the aggregation ability of SAP in different environments, 250 μg/mL SAP, the highest test concentration was introduced into *E. coli* ATCC 25922 culture (10^8^ CFU/mL) prepared in an EtOH-sterilized cuvette. As shown in Additional file 1: Figure S5, bacterial clumping was observed under three pH conditions after adding SAP, and these clumps sedimented to the bottom of the cuvette within 8 h. In particular, the supernatant of the bacterial solution was substantially clear after 3 h at pH 6.0, and SAP somewhat reduced the turbidity of the bacterial supernatant at pH 5.0 but not as fast as that at pH 6.0 (Additional file 1: Figure S5a, b). In a neutral environment (pH = 7.0), SAP could entrap bacteria to form milky bacterial solutions, indicating that peptides and bacteria quickly form a higher degree of aggregation (Additional file 1: Figure S5c). From the results of the change in bacterial numbers in the supernatant, the sedimentation rate of bacteria was the fastest in pH neutral solution, and the number of colonies was significantly decreased from 10^8^ CFU/mL to 10^2^ CFU/mL within 3 h, followed by pH = 6.0 from 10^8^ CFU/mL to zero within 8 h (Fig. 4b). In contrast, agglutination and sedimentation were not observed for the untreated control. These results revealed that the pH-triggered self-assembled peptide SAP has the ability to recognize and trap bacteria, and the higher the pH was, the faster the bacteria clumped. To determine whether the bacterial sedimentation was due to the automatic sedimentation of the peptide by changing pH, 250 μg/mL SAP was added into 4-(2-hydroxyethyl) piperazine-1-ethanesulfonic acid (HEPES) buffer (pH = 5.0, 6.0 and 7.0) for 8 h. Indeed, incubating the SAP without *E. coli* did not aggregate as previously identified after the addition of *E. coli*, indicating that the bacterial aggregation was the result of the interaction between peptides and bacteria (Additional file 1: Figure S6).

Considering the antimicrobial and agglutination properties, pH = 6.0 was selected as the model environment to investigate the bacterial agglutination efficiency of different SAP concentrations (3.9–250 μg/mL). As shown in Additional file 1: Figure S7, at SAP concentrations above 62.5 μg/mL, discernible formation of aggregation could be observed. In particular, the formation of aggregation was observed within 0.5 h at peptide concentrations of 250 μg/mL and 125 μg/mL, and at peptide concentration of 62.5 μg/mL, the formation of aggregation was observed within 1 h. At concentrations below 31.2 μg/mL, no significant bacterial aggregation was observed, even though the peptide showed antibacterial activity above 15.6 μg/mL. This was also illustrated by the results of the change in bacterial numbers in the supernatant, and a significant decrease was observed in the number of colonies when the peptide concentration was above 62.5 μg/mL. However, no aggregation phenomenon was observed in the concentration of peptide in the range of 15.6 μg/mL to 31.2 μg/mL. Subsequently, bacterial aggregation mediated by SAP was observed using a fluorescence microscope. After treatment with 250 μg/mL SAP, *E. coli* particle aggregation and internalization of nucleic acid stain PI into *E. coli* was observed, that determined SAP could induce bacterial aggregation and accompanying bacterial death (Fig. 4d).

This bacteria-entrapped function of peptides was first reported by Bevins and co-workers in 2012, they seminally investigated that the human α‑defensin 6 (HD6) undergoes ordered self-assembly into fibrils and nanonets to entrap and agglutinate *Salmonella Typhimurium* to prevent human gastrointestinal pathogens from invading intestinal mucosal cells, rather than kill bacteria directly [22]. This mechanism is also observed in neutrophil extracellular traps (NETs), an extracellular web-like neutrophil-derived protein network, which plays a role to trapping invading bacteria such as *Klebsiella pneumoniae* and inducing a series of immunoreaction [42]. Focused on the host defense model of HD6, Phoom and co-workers investigated the molecular basis of self-assembly behavior and capacity of entrapping bacteria via site-directed mutagenesis [24]. Absent of bacteria and other biomolecules, four HD6 monomers could form a hydrophobic pocket and the reciprocity of these tetramers promote the formation of fiber chains in the aqueous buffer [43]. Among these reciprocities, the hydrophobic interaction provided by Phe2 and Phe29 is essential for the self-assembly ability and the Phe2Ala and Phe29Ala variants caused the depolymerization of HD6 and failed to prevent *L. monocytogenes* invasion, which indicates that the self-assembly is important for microbial entrapment [24]. The experimental results of this study further verified this point. The SAP could trap *E. coli* at a concentration of 62.5 μg/mL, this was above the CAC under three environments. In the neutral, the higher turbidity was observed in the bacterial solution due to the higher aggregation degree and instant formation of larger bacterial clusters, and that was because SAP was in a neutral status, and the hydrophobic force of Val and Trp mediates the interaction between peptide and bacteria, inducing a rearrangement of peptides, promoting the agglutination of bacteria via aggregation of surface-attached peptide molecules [21, 44]. Moreover, different bacteria seem to be developed different nanonet structures and the amount of nanonets wrapping via microscopic analysis [45]. And Chu and co-workers investigated that the interactions exist between the HD6 nanonets and the surface protein of *S. Typhimurium*. These evidences indicate that this entrapment seem to be mediated by the interactions between peptide and bacterial surface protein or other components, and Yu and co-workers determined that this possibility is feasible. They introduced a ligand peptide, which could specially bind to lipoteichoic acid (LTA), into the self-assembly system and the specific binding to LTA mediated the transformation of nanoparticles into nanorods, accompanied by the agglutination of *S. aureus* [23]. As for our study, further studies are needed to determine whether SAP-mediated bacterial agglutination is related to peptide-receptor interactions. In addition, Lim and co-workers also attributed this bacterial-peptide aggregation phenomenon to ligand-receptor interactions and pointed out that the length of nanofibers had a significant influence on the formation efficiency of bacterial clusters. The longer fibers tended to promote agglutination of large bacterial clusters, which seemed to have more bacterial binding sites, but the short nanofibers seemed to interconnect a single or small number of bacteria [46]. And our results also confirmed this point that longer fibers could cause higher agglutination degree of *E. coli*. It is noteworthy that in our study, the SAP had antimicrobial activity in addition to its trapping function. Marc and co-workers reported that the aggregation process triggered the subsequent membrane permeabilization [21], but our results were somewhat at odds with this point. The SAP-mediated bacterial agglutination degree observed in acidic conditions was weaker than in neutral conditions, but bacterial aggregation process should not exclude the possibility that parts of bacteria were killed directly by the peptide. The antimicrobial activity of peptide is determined by a positive charge and hydrophobicity of peptide, while the trapping process is mediated by hydrophobic interactions between peptide and bacterial surface. The possibility of these two processes occurring simultaneously cannot be excluded, but they are independent of each other.

Subsequently, we hypothesized that bacterial agglutination could promote the effective clearance of bacteria particles of phagocytes at bacteria-infected sites. To test this, the uptake of *E. coli* particles by RAW 264.7 was measured using 3D-SIM super-resolution microscopy. As shown in Fig. 4e, *E. coli* particles, aggregated by SAP, increased the internalization of RAW 264.7, compared with control, which showed limited phagocytic activity of RAW 264.7 cells. The result indicated that SAP was able to aggregate bacteria and promoted phagocytic uptake, which played an important role in clearance of *E. coli* by phagocytes.

### In-vivo efficacy and biocompatibility of SAP

**Fig. 5:**
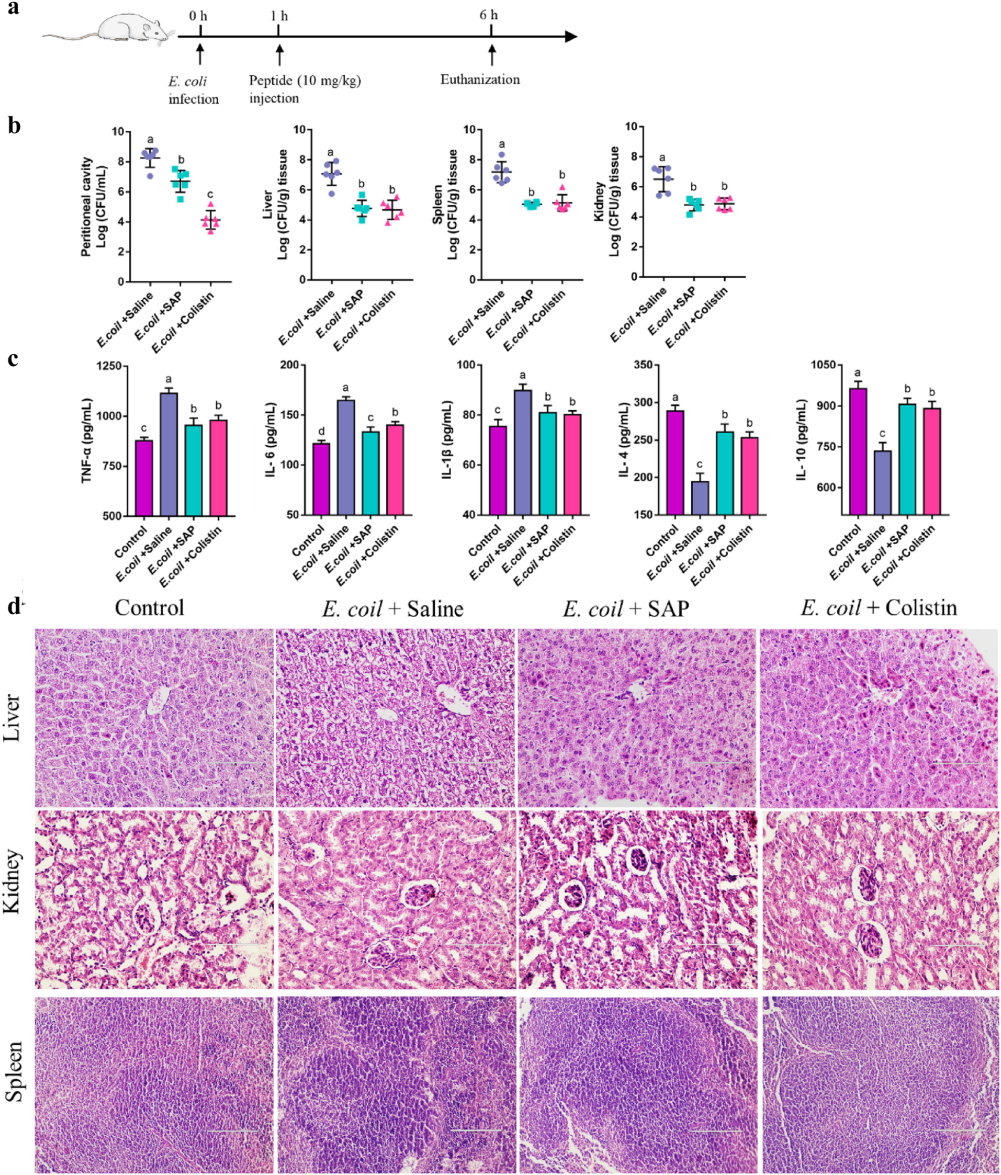
**a** Schematic illustration of the experimental protocol for efficacy measurement. **b** Bacterial burdens in liver, spleen, kidney and peritioneal cavity after infection of *E. coli* and treatment with saline, SAP and colistin (mean ± SD, n = 6, *p* < 0.05). **c** The levels of TNF-α, IL-6, IL-1β, IL-4 and IL-10 after injection of saline and infection of *E. coli* and treatment with saline, SAP and colistin (mean ± SD, n = 6, *p* < 0.05). **d** Histopathological H&E staining of liver, spleen and kidney tissue after injection of saline and infection of *E. coli* and treatment with saline, SAP and colistin

The above results of bacterial agglutination assay indicated that SAP showed the best entrapment activity in the neutral environment. For further evaluate in vivo therapeutic efficacy of SAP, a peritonitic-bacteremic mice model was implemented to explore the entrapment ability in vivo to prevent bacteria from invading the animal tissues. As shown in Fig. 5a, each mouse in the infection group was intraperitoneally injected with 1.5 × 10^8^ CFU/mL *E. coli* ATCC 25922 and each mouse in the control group was injected with the same amount of saline. At 1 h postinfection, each mouse in the treatment group was intraperitoneally injected with 10 mg/kg SAP and 1 mg/kg colistin. Compared with control, bacterial burdens in the liver, spleen, kidney and peritoneal cavity were significantly reduced after the treatment of SAP and colistin (*p* < 0.05), and there are no significant differences only in organs between SAP and colistin treatment (*p* > 0.05) (Fig. 5b), which indicated that the acidic peptide initially exhibited antimicrobial activity like colistin, resulting in a decrease in the enumeration of bacterial colonies in the blood and the peritoneal cavity, and then the peptides translated to a neutral state that entrapped bacteria and prevented them from invading the tissue. Moreover, the levels of proinflammatory cytokine TNF-α, IL-6, and IL-1β of SAP treatment were significantly lower compared with the *E. coli* + saline group (*p* < 0.05), and the levels of anti-inflammatory cytokine IL-4 and IL-10 of SAP treatment were significantly higher compared with the *E. coli* + saline group (*p* < 0.05) and no significant differences were noted compared colistin treatment (*p* > 0.05) (Fig. 5c). The H&E staining showed that the treatment of SAP and colistin alleviated *E. coli*-induced tissue damage, such as hepatocytes damage and loss of the nucleus and cytoplasm, swelling of the glomerulus and lymphocyte infiltration in the spleen, which promoted liver cell regeneration, normalization of glomerular structures and spleen tissues (Fig. 5d). These data indicated that SAP could attenuate the bacteremia-induced inflammatory cytokine levels and organ tissue damage via killing and entrapping bacteria and regulating the body’s immune system [47]. In a word, in-vivo and in-vitro biological activities, biocompatibility and efficacy highlighted the promoting potential of pH-triggered β-hairpin forming nanopeptide for the treatment of acid-tolerant enteric pathogens induced infection.

**Fig. 6:**
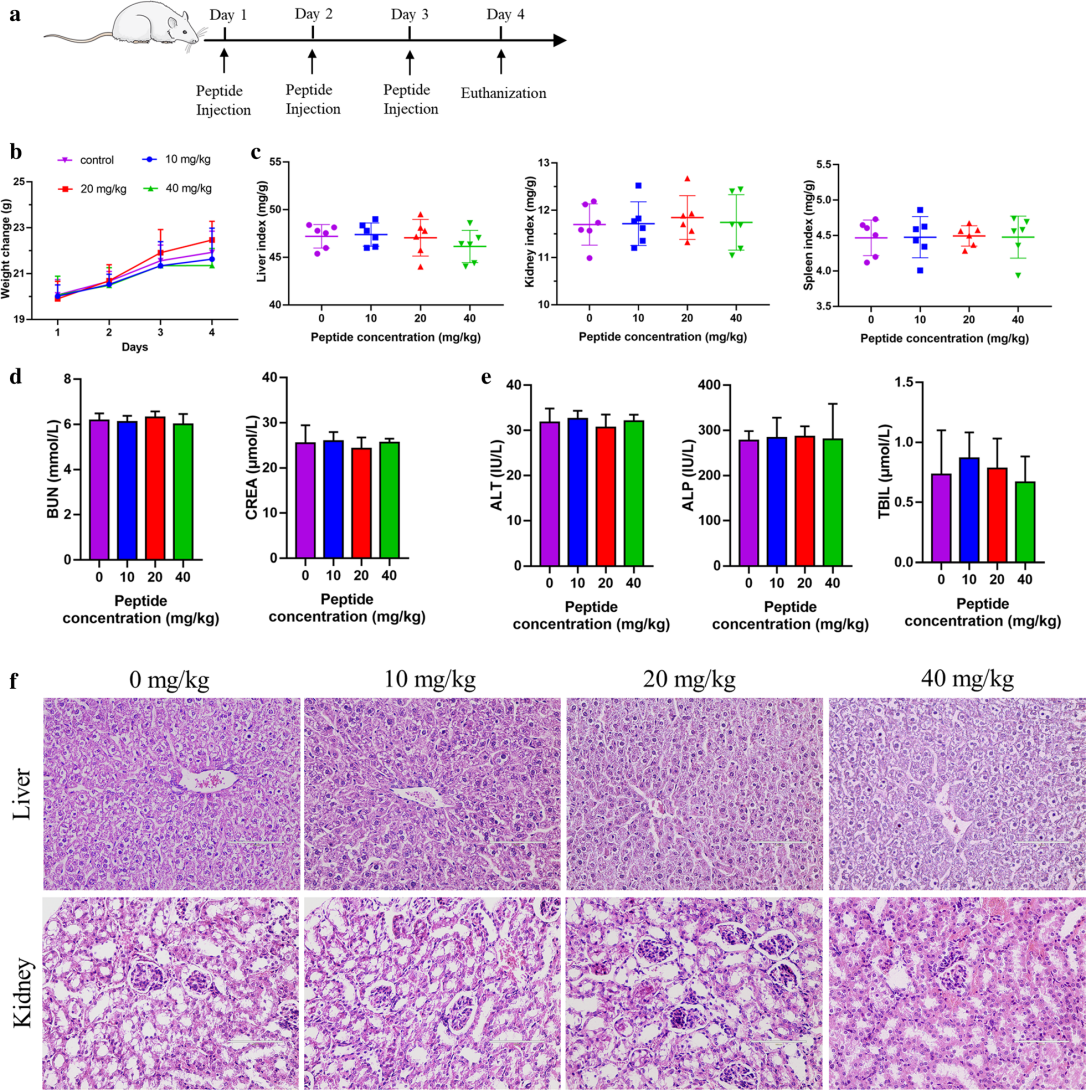
**a** Schematic illustration of the experimental protocol for biocompatibility measurement. **b** Curve of weight change, **c** relative organ index, **d** renal function parameters and **e** hepatic function parameters of mouse injected with 10 mg/kg, 20 mg/kg, 40 mg/kg and saline in four days (mean ± SD, n = 6, *p* > 0.05). **f** Histopathological H&E staining of liver and kidney tissue after injection of SAP and saline

For further investigation of the safety of SAP in vivo, 6 mice in each group were injected intraperitoneally with 10 mg/kg, 20 mg/kg, 40 mg/kg SAP, and body weight and relative organ weight were monitored to assess the toxicity of SAP and then serum was taken out to measure serum alanine aminotransferase (ALT), alkaline phosphatase (ALP), total blood bilirubin (TBIL) activity, blood urea nitrogen (BUN) and serum creatinine (CRAE) for assessment of the hepatotoxicity and nephrotoxicity, respectively [48]. As shown in Fig. 6, there were no significant differences in the body weight (Fig. 6b), and the liver, kidney and spleen index (Fig. 6c) of all groups. Meanwhile, no significant differences were noted on the levels of ALT, ALP, TBIL, BUN and CRAE between the control group and peptide-injected groups (Fig. 6d and e). Additionally, histological examination (H&E) also showed that there are no obvious tissue abnormalities of all treatment groups (Fig. 6f). Taken together, SAP have good biocompatibility, resulting in a wide range of development and application potentials.

## Conclusions

In summary, a novel pH-triggered self-assembled β-hairpin nanopeptide SAP was successfully designed as an effective drug with antibacterial and entrapping dual functions. The pH-induced change in the secondary structure from random coil structures at pH 5.0 to β-sheets at pH 7.0 resulted in morphological evolution of the microstructure from nanospheres to nanofibers, achieving the aim of switching its biological activity ‘on’ and ‘off’ in response to changes in pH. Moreover, the nanopeptide at high concentrations could induce bacterial aggregation, which may contribute to the clearance of bacteria at the infection site. Meanwhile, SAP proven to be excellent therapeutic effect in the peritonitic-bacteremic mouse model and great biocompatibility in vivo and in vitro. This study revealed the conformational change of β-hairpin forming nanopeptides with changes in pH conditions and their biological functions, providing an innovative design strategy and antibacterial mechanism to target specific acid resistance mechanisms in the case of a pathogenic bacterial infection.

## Experiment section

### Bacterial strains, growth conditions and materials

The gram-negative strains *Escherichia coli* ATCC 25922, *Salmonella typhimurium* ATCC 14028 and the gram-positive strain *Listeria monocytogenes* CGMCC 1.10753, *Bacillus cereus* CGMCC 1.932 were obtained from the School of Veterinary Medicine, Northeast Agricultural University (Harbin, China). The bacterial growth medium was tryptic soy broth/agar (TSB/A), which was obtained from Hopebio-Technology Co., Ltd. 1-Anilino-8-naphthalene sulfonate (ANS, > 97%) was purchased from Sigma-Aldrich (Oakville, Canada). Dimethyl formamide (DMF, > 99.9%) was purchased from Aladdin.

### Sample preparation

The peptide SAP (Trp Val His His Trp Val His His Trp Val His His *pro* Gly His His Val Val His His Val Val His His Val Val-NH_2_) in this study was synthesized by Sangon Biotech Co., Ltd. (Shanghai, China), and the molecular masses were analyzed using matrix-assisted laser desorption/ionization time-of-flight mass spectrometry (MALDI-TOF MS; Linear Scientific, Inc., USA) using α-cyano-4-hydroxycinnamic acid as the matrix. Lyophilized powders were dissolved to different concentrations in Milli-Q water, sonicated for 30 min to reach a monomeric state and then allowed to self-assemble at room temperature for 6–7 days. The pH of the peptide solution was adjusted using hydrogen chloride solution or sodium hydroxide solution and measured by a pH5S Spear pH Tester.

### ANS fluorescence assay

The critical assembly concentration (CAC) of SAP was determined using the 1-anilino-8-naphthalene sulfonate (ANS) fluorescence assay. ANS was dissolved in DMF to a concentration of 1 mM, and then 1 µL of ANS was added to 100 µL of different concentrations of nanopeptide. The mixed solution was transferred to a quartz cuvette and tested using an F-4500 fluorescence spectrophotometer (Hitachi, Japan), and the fluorescence spectrum was collected from 420 to 670 nm with excitation at 360 nm. The CAC was determined from the matched curve, which was obtained by the ANS fluorescent intensities at 475 nm plotted against different concentrations of peptide.

### Transmission electron microscopy (TEM) of peptide samples

To characterize the shape of peptide in neutral and acidic environments, SAP was visualized using transmission electron microscopy (HITACHI HT7800 TEM). Ten microliters of peptide solution were plated on copper-coated grids (400 square mesh, Electron Microscopy Sciences) for 5 min, and the grids were stained with 0.1% phosphotungstic acid for 30 s and dried for 15 min before imaging, operating at 100 kV.

### Circular dichroism spectroscopy (CD)

The CD spectra of SAP were recorded on a J-820 spectropolarimeter (Jasco, Tokyo, Japan) at a final concentration of 0.5 mg/mL (153 µM) in Milli-Q water (pH = 5.0, 6.0 and pH = 7.0). Data were collected from 195 to 260 nm at room temperature three times using a quartz cell with a 1.0 mm path length. The acquired CD spectra were then converted to the mean residue ellipticity by using the following equation:

$$\theta _{{\text{M}}} = \left( {\theta _{{{\text{obs}}}} *{\text{1}}000} \right)/\left( {{\text{c}}*{\text{n}}*{\text{l}}} \right)$$

where c is the concentration of peptide solution (µM), n is the number of amino acids of peptide, and l is the path length (nm).

### Measurement of the hydrodynamic diameter of the particles

The hydrodynamic diameter of the particles present in the peptide-ellipticine formulation was obtained using a Zetasizer Nano Z90 (Malvern Instruments, Worcestershire, U.K.). Formulated solutions (1 mL) were placed in a disposable plastic cuvette. The reflective index and viscosity of pure water were used as the solvent settings, while the reflective index of the solute was set to be the same as that of the proteins. The temperature was maintained at 25 °C, and the solution was equilibrated for 2 min before data acquisition. Three measurements were performed for each sample. The data were automatically analyzed with the software to generate the intensity-based size distribution of the complexes.

### Assessment of bacterial activity

The antibacterial activity of peptides was investigated by the viable count assay. A single colony was isolated from an agar plate, cultured in 5 mL of 3% tryptic soy broth (TSB) medium and shaken at 200 rpm overnight at 37 °C. Fifty microliters of bacterial suspension was added to 5 mL of fresh 3% TSB and shaken at 200 rpm until logarithmic phase, which was diluted to approximately (2–5) × 10^5^ CFU/mL with fresh TSB and mixed with peptide at different concentrations from 3.9 to 250 µg/mL. After incubation at 37 °C for 3 h, serial dilutions of samples were plated on TSB agar and then incubated at 37 °C overnight. To determine the effect of NaCl on the bacterial activity of peptides, 100 mM and 150 mM NaCl were mixed into TSB with peptides at different concentrations. Protease resistance of the peptide was assessed as described above except preincubation of peptide and enzymes (trypsin and chymotrypsin) at mass ratios of 2:1, 1:1, 0.5:1 and 0.25:1 (enzyme: peptide), respectively, for 1 h at 37 °C.

### Measurement of cytoplasmic membrane electrical potential

The cytoplasmic membrane depolarization induced by the SAP was measured by the membrane potential-sensitive fluorescent dye diSC_3-5_ (Sigma-Aldrich) as previously described [49]. Briefly, logarithmically growing *E. coli* ATCC 25922 cells were harvested by centrifugation at 1000×*g* for 5 min and resuspended in 5 mM HEPES buffer (pH 6.0, containing 20 mM glucose). Two milliliters of bacterial suspension were incubated with 0.4 μM diSC_3-5_ and 100 mM K^+^ mixed with peptides at different concentrations. The fluorescence was continuously detected for 3300 s (excitation λ = 622 nm, emission λ = 670 nm) with an F-4500 fluorescence spectrophotometer (Hitachi, Japan).

### Scanning electron microscopy (SEM) of bacterial samples

*E. coli* ATCC 25922 was cultured in TSB overnight and reactivated in new TSB to a logarithmic period. The cells were centrifuged at 1000×*g* for 5 min, washed three times with PBS (10 mM, pH = 6.0) and then diluted to an OD_600_ of 0.2. Peptide (250 μg/mL) was incubated with the bacteria at 37 °C for 1 h and harvested by centrifugation. The bacterial samples were fixed with 2.5% (w/v) glutaraldehyde at 4 °C overnight, dehydrated with a graded series of ethanol (50, 70, 90 and 100%) and then added to a mixture (v:v = 1:1) of alcohol and tert-butanol for 30 min and tert-butanol alone for 1 h. The specimens dried by using a critical point dryer were coated and visualized under a field emission scanning electron microscope (Hitachi S-4800, Japan).

### Transmission electron microscopy (TEM) for bacterial samples

*E. coli* ATCC 25922 was incubated as described in “Scanning electron microscopy (SEM) of bacterial samples” section. Briefly, harvested bacteria were incubated with 250 μg/mL peptide for 1 h and centrifuged to obtain treated bacteria before fixation with 2.5% glutaraldehyde. The next day, the samples were postfixed with 2% osmium tetroxide for 70 min, continuously dehydrated with a graded ethanol series (50%, 70%, 90%, and 100%) for 8 min and a mixture (v:v = 1:1) of 100% ethanol, acetone for 15 min and absolute acetone for 15 min. Subsequently, the bacterial samples were immersed in a 1:1 mixture of absolute acetone and epoxy resin for 30 min and pure epoxy resin overnight. Finally, treated bacterial morphology was determined using a HITACHI H-7650 TEM.

### Measurement of cytotoxicity

The cytotoxicity of the nanopeptide was determined with two mammalian cell lines, including the murine macrophage cell line RAW264.7 and human embryonic kidney (HEK) 293T cells, by using a 3-(4,5-dimethylthiozol-2-yl)-2,5-diphenyltetrazolium bromide (MTT) dye reduction assay. RAW264.7 and HEK 293T cells were cultured in Dulbecco’s modified Eagle medium (DMEM) supplemented with 10% heat-inactivated FBS and 1% penicillin/streptomycin. In the cytotoxicity assay, (1.0–2) × 10^5^ cells/well were plated into 96-well plates and incubated for 24 h at 37 °C in 5% CO_2_, and the supernatant medium was removed. Then, cells were challenged with peptides at different concentrations and incubated under a humidified atmosphere of 5% CO_2_ overnight. After incubating for 4 h, 50 μL of 5 mg/mL MTT (dissolved in sterile PBS) was added to the cultures for 4 h at 37 °C in 5% CO2, and then the supernatants were discarded. The formazan crystals were dissolved in 150 μL of dimethyl sulfoxide (DMSO). The absorbance of the solution was measured at 570 nm using a microplate reader (TECAN GENios F129004; TECAN, Austria). The assay was repeated at least three times. The cell viability was obtained using the following equation:

$${\text{Cellviability}}\left( \% \right) = \left[ {\left( {{\text{A}} - {\text{A}}_{0} } \right)/\left( {{\text{A}}_{{\text{t}}} - {\text{A}}_{0} } \right)} \right] \times {\text{1}}00\% ,$$

where A represents the absorbance of the peptide sample at 570 nm, A_0_ represents the absorbance of the negative control and A_t_ represents the absorbance of the positive control. The results presented are the mean of three independent experiments conducted ± the standard error of the mean (SD).

### Measurement of hemolysis assay

The hemolysis activity of the nanopeptide was measured as the amount of hemoglobin released by the lysis of erythrocytes as described previously [27]. Fresh blood was collected by cardiac puncture of the left ventricle from female Institute for Cancer Research (ICR) mice (SPF, 20–22 g) sacrificed by excessive anesthesia administration. Fresh blood was washed three times, and erythrocytes were resuspended in 10 mM PBS (pH 7.4 or 6.0). Fifty microliters of fresh blood cell suspension were mixed with 50 μL of different concentrations of peptide solution in a 96-well plate and incubated at 37 °C for 1 h. Then, the challenge plate was centrifuged at 1000×*g* for 5 min at 4 °C, and the supernatant was carefully removed into a new 96-well plate. The released hemoglobin was recorded by the absorbance of the supernatants at 570 nm with a microplate reader (Tecan, Austria). Erythrocytes in PBS and 0.1% Triton X-100 were measured as negative and positive controls, respectively. Hemolysis was obtained using the following equation:

$${\text{Hemolysis }}\left( \% \right) = \left[ {\left( {{\text{A}} - {\text{A}}_{0} } \right)/\left( {{\text{A}}_{{\text{t}}} - {\text{A}}_{0} } \right)} \right] \times {\text{1}}00\% ,$$

where A represents the absorbance of the peptide sample at 570 nm, A_0_ and A_t_ represent the absorbance of the negative control and positive control, respectively. The results presented are the mean of three independent experiments conducted ± the standard error of the mean (SD).

### Bacterial agglutination assays

*E. coli* ATCC 25922 was incubated as described in “Assessment of bacterial activity” section, followed by centrifugation at 1000×*g* for 5 min and washing three times with HEPES buffer (pH = 7.0). The obtained bacteria were resuspended in HEPES buffer (pH = 5.0, 6.0 and 7.0) to an OD_600_ value of 0.25. Different concentrations of the peptide were mixed with bacterial solution in an EtOH-sterilized cuvette and incubated for 8 h at room temperature. At every time point, a 50 μL aliquot from each cuvette was diluted with 450 μL of 11 mM sodium phosphate pH 7.4, vortexed gently, serially diluted from 10^−2^ to 10^−7^ in tenfold increments and plated on TSB agar for incubation at 37 °C overnight.

### 3D-SIM super-resolution microscopy imaging

*E. coli* strain BL21 consisting of PET-28a-EGFP plasmid was used to investigate the bacterial agglutination behavior induced by SAP. Briefly, *E. coli* strain BL21 was cultivated in LB (Sigma Aldrich) containing 50 μg/mL kanamycin at 37 °C and added 0.5 mM isopropyl β-d-thiogalactoside (IPTG) at 30 °C overnight to induced EGFP protein expression. *E. coli* particles were harvested by centrifugation at 1000×*g* for 5 min and resuspended in 5 mM HEPES buffer (pH 6.0, containing 20 mM glucose). 250 μg/mL SAP was added into bacterial solution (OD_600_ = 0.4) for 1 h at 37 °C and harvested *E. coli* particles by centrifugation. 10 μg/mL PI dye was added and incubated for 15 min at 4 °C and the unbound PI dye was removed by washed three times with HEPES buffer. The sample was smeared on the glass slide and observed using Deltavision OMX SR fluorescence microscope.

RAW 264.7 cells [(1.0–2) × 10^5^ cells/well] were incubated on confocal petri dish overnight for adherence. *E. coli* was harvested incubated as described above and harvested by centrifugation at 1000×*g* for 5 min and preincubated with 250 μg/mL SAP in 5 mM HEPES buffer (pH 6.0) for 1 h at 37 °C. The pretreated *E. coli* suspension was added into cell culture and infected RAW 264.7 cells for 2 h at 37 °C. After removing the *E. coli*, the cells were fixed with immunol staining fix solution (Beyotime, China) and immunostaining permeabilization solution with Triton X-100 (Beyotime, China) for 10 min, respectively and treated with antifade mounting medium with DAPI. The samples were observed under a fluorescence microscope (Deltavision OMX SR).

### In-vivo efficacy assessment

Twenty-four female ICR mice (6–8 weeks old, 20–22 g) were randomly divided into four groups (6 per cage): control; *E. coli* + saline; *E. coli* + SAP; *E. coli* + colistin. After 3 days pre-feeding, 200 μL saline (control) and 1.5 × 10^8^ CFU/mL *E. coli* (*E. coli* + saline; *E. coli* + SAP; *E. coli* + colistin) were intraperitoneally injected. After 1 h, saline (control; *E. coli* + saline), 10 mg/kg SAP (*E. coli* + SAP), and 1 mg/kg colistin (*E. coli* + colistin) were intraperitoneally injected following Shao and co-workers reported [48]. After 6 h, the mice were sacrificed by excessive anesthesia administration and the serum was collected for evaluation of the levels of the inflammatory cytokines. 2 mL saline was injected into every mouse and the peritoneal cavity fluids were harvested, diluted with sterile saline and plated on the TSA agar for measurement of bacterial colonies. The organ homogenate (liver, spleen and kidney) was harvested and plated on TSA for the bacterial count. The liver and kidney were fixed using 10% formalin for H&E staining.

### In-vivo toxicity assessment

Twenty-four female ICR mice (6–8 weeks old, 20–22 g) were purchased from Liaoning Changsheng Biotechnology Company and obtained enough standardized basal diet and deionized water. On the first day of the experiment, mice were randomly divided into four groups (6 per cage), and intraperitoneally injected with saline and 10 mg/kg, 20 mg/kg, and 40 mg/kg SAP every 24 h for 3 days. The bodyweight of mice was monitored every day. On the fourth day, the mice were sacrificed by excessive anesthesia administration and the serum was collected for evaluation of the levels of serum alanine aminotransferase (ALT), alkaline phosphatase (ALP), total blood bilirubin (TBIL) activity, blood urea nitrogen (BUN) and serum creatinine (CRAE) with an automatic biochemical analyzer (Roche Cobus Mira Plus, Switzerland). The weight of the liver, kidney, and spleen was measured and the liver and kidney were fixed using 10% formalin for H&E staining.

### Statistical analysis

Data are shown as the mean ± standard deviation (SD), analyzed by unpaired Student’s t-test or one-way analysis of variance (ANOVA) with the following *p* values: * *p* < 0.05, ** *p* < 0.01, *** *p* < 0.001.

## Supplementary Information

**Additional file 1: Figure S1.** HPLC of the pH-triggered self-assembled β-hairpin self-assembled peptide (SAP).** Figure S2.** MALDI-TOF MS spectrum of the pH-triggered self-assembled β-hairpin self-assembled peptide (SAP).** Figure S3.** (a) Thioflavin T (ThT) fluorescence assay of SAP at pH 5.0, 6.0 and 7.0. (b) Hydrodynamic size of 15.6 μg/ml SAP at pH 5.0. (c)ζ potential of SAP at pH 5.0, 6.0 and 7.0.** Figure S4. **Antibacterial activity of the SAP against* E. coli *ATCC 25922 in presence of 100 mM and 150 mM NaCl at pH 5.0 (mean ± SD, n = 3) (*p* < 0.05).** Figure S5.** Bacterial agglutination assay for the SAP. Agglutination of *E. coli *ATCC 25922 treated with 250 μg/mL SAP for 8 h in HEPES buffer at pH=5.0 (a), 6.0 (b) and 7.0 (c).** Figure S6.** Agglutination assay without bacteria as a control. SAP (250 μg/mL) was added to HEPES buffer at pH=5.0 (a), 6.0 (b) and 7.0 (c).** Figure S7. **Bacterial agglutination assay for different concentrations of SAP. Agglutination of *E. coli *ATCC 25922 treated with 0 μg/mL (a), 250 μg/mL (b), 125 μg/mL (c), 62.5 μg/mL (d), 31.5 μg/mL (e), 15.6 μg/mL (f), 7.8 μg/mL (g) and 3.9 μg/mL (h) SAP for 8 h in HEPES buffer at pH=6.0.

## Data Availability

All data and materials used are all available in the manuscript. All data generated or analyzed during this are included in this published article.
