# Supplementary material for: De novo design of a pH-triggered self-assembled β-hairpin nanopeptide with the dual biological functions for antibacterial and entrapment
Source: J Nanobiotechnology. 2021 Jun 14;19:183. doi: 10.1186/s12951-021-00927-z (PMC8201815; doi:10.1186/s12951-021-00927-z)
Supplement: Supplementary file 1 — Additional file 1: Figure S1. HPLC of the pH-triggered self-assembled β-hairpin self-assembled peptide (SAP). Figure S2. MALDI-TOF MS spectrum of the pH-triggered self-assembled β-hairpin self-assembled peptide (SAP). Figure S3. (a) Thioflavin T (ThT) fluorescence assay of SAP at pH 5.0, 6.0 and 7.0. (b) Hydrodynamic size of 15.6 μg/ml SAP at pH 5.0. (c)ζ potential of SAP at pH 5.0, 6.0 and 7.0. Figure S4. Antibacterial activity of the SAP against E. coli ATCC 25922 in presence of 100 mM and 150 mM NaCl at pH 5.0 (mean ± SD, n = 3) (p < 0.05). Figure S5. Bacterial agglutination assay for the SAP. Agglutination of E. coli ATCC 25922 treated with 250 μg/mL SAP for 8 h in HEPES buffer at pH=5.0 (a), 6.0 (b) and 7.0 (c). Figure S6. Agglutination assay without bacteria as a control. SAP (250 μg/mL) was added to HEPES buffer at pH=5.0 (a), 6.0 (b) and 7.0 (c). Figure S7. Bacterial agglutination assay for different concentrations of SAP. Agglutination of E. coli ATCC 25922 treated with 0 μg/mL (a), 250 μg/mL (b), 125 μg/mL (c), 62.5 μg/mL (d), 31.5 μg/mL (e), 15.6 μg/mL (f), 7.8 μg/mL (g) and 3.9 μg/mL (h) SAP for 8 h in HEPES buffer at pH=6.0. [file 12951_2021_927_MOESM1_ESM.docx]

Additional Information

***De novo* design of a pH-triggered self-assembled β-hairpin nanopeptide with the dual biological functions for antibacterial and entrapment**

Qiuke Li†, Jinze Li†, Weikang Yu, Zhihua Wang, Jiawei Li, Xingjun Feng, Jiajun Wang*,Anshan Shan*

Laboratory of Molecular Nutrition and Immunity. The Institute of Animal Nutrition, Northeast Agricultural University, Harbin, P. R. China.

^*^Corresponding author: Anshan Shan, [asshan@neau.edu.cn](mailto:asshan@neau.edu.cn); Jiajun Wang, wjj1989@neau.edu.cn

†These authors contributed equally to this work.


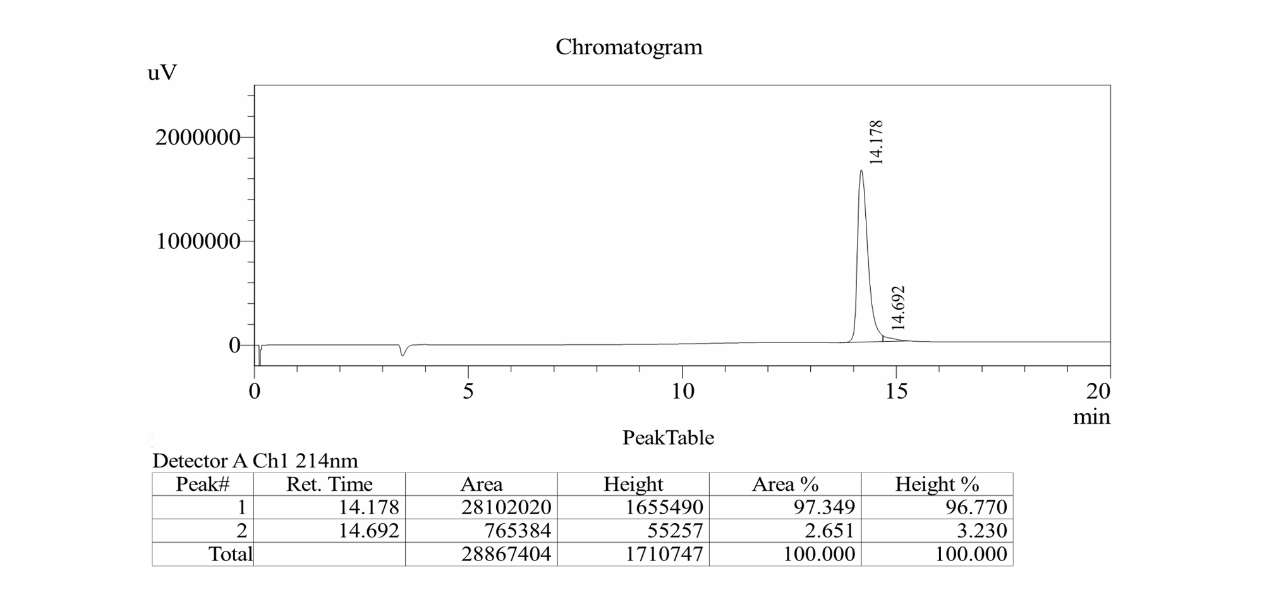


**Figure S1** HPLC of the pH-triggered self-assembled β-hairpin self-assembled peptide (SAP).


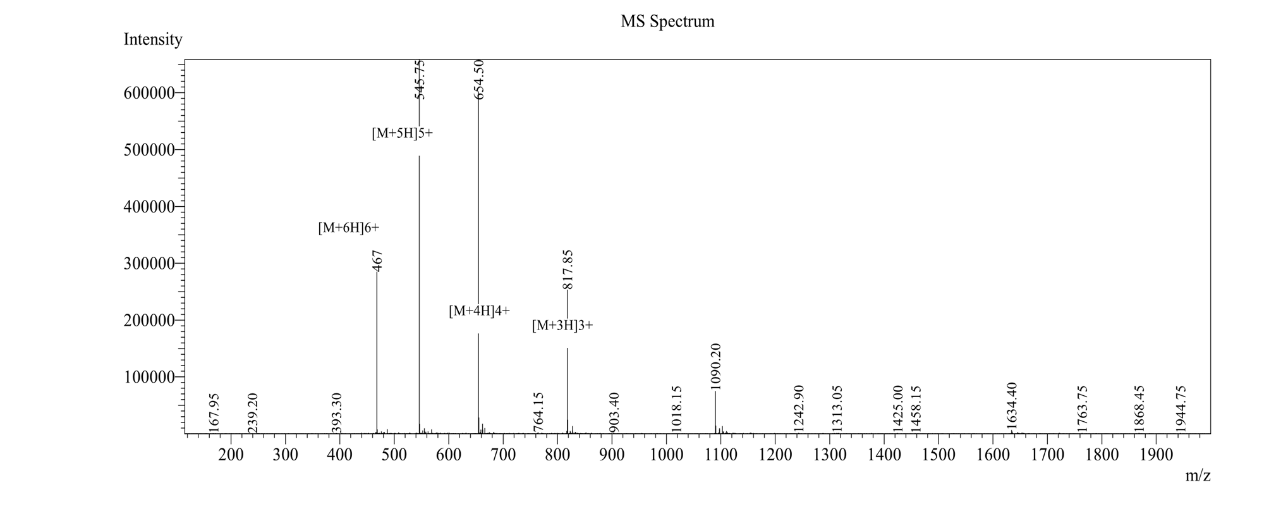


**Figure S2** MALDI-TOF MS spectrum of the pH-triggered self-assembled β-hairpin self-assembled peptide (SAP).


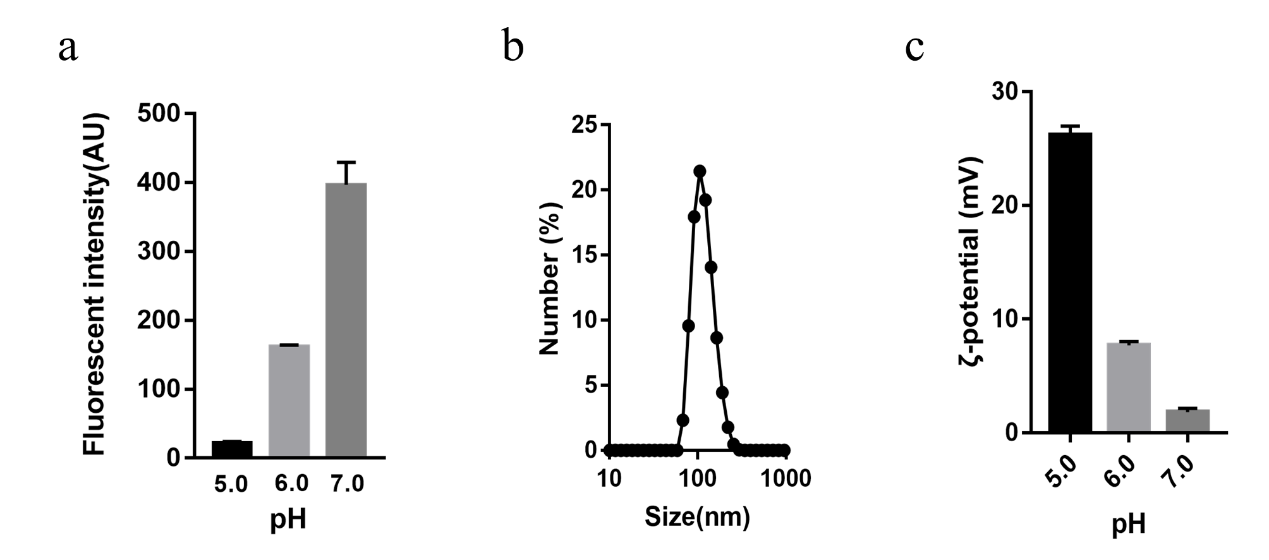


**Figure S3** (a) Thioflavin T (ThT) fluorescence assay of SAP at pH 5.0, 6.0 and 7.0. (b) Hydrodynamic size of 15.6 μg/ml SAP at pH 5.0. (c)ζ potential of SAP at pH 5.0, 6.0 and 7.0.


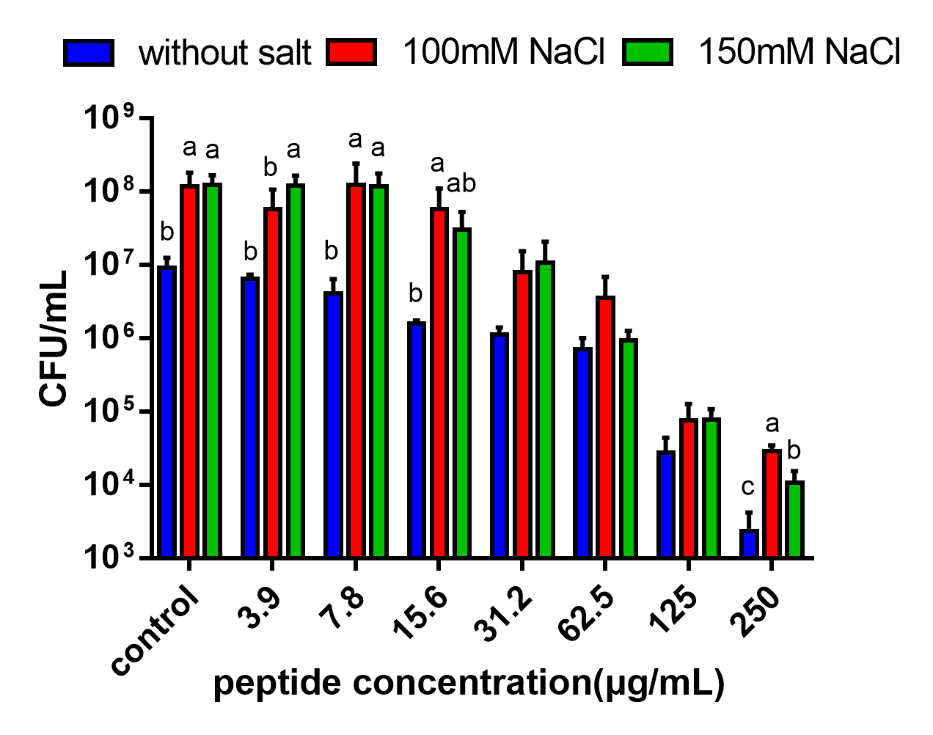


**Figure S4** Antibacterial activity of the SAP against *E. coli* ATCC 25922 in presence of 100 mM and 150 mM NaCl at pH 5.0 (mean ± SD, n=3) (*p*<0.05).


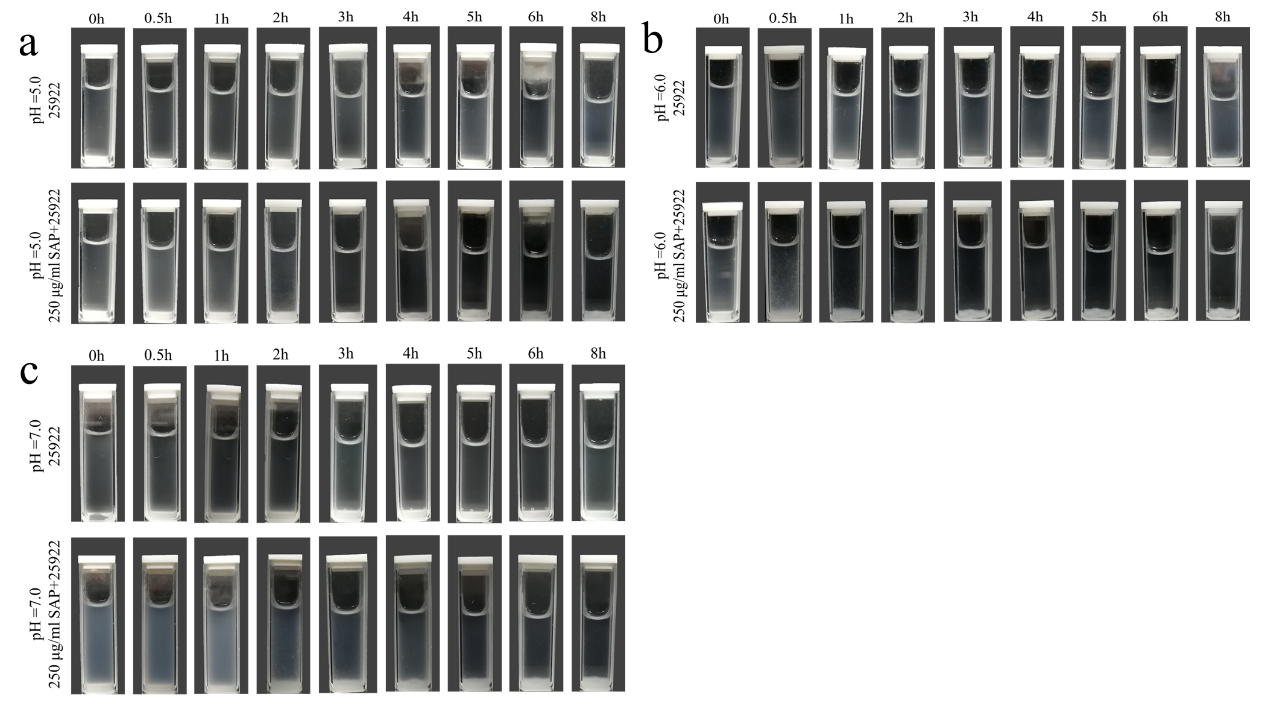


**Figure S5** Bacterial agglutination assay for the SAP. Agglutination of *E. coli* ATCC 25922 treated with 250 μg/mL SAP for 8 h in HEPES buffer at pH=5.0 (a), 6.0 (b) and 7.0 (c).


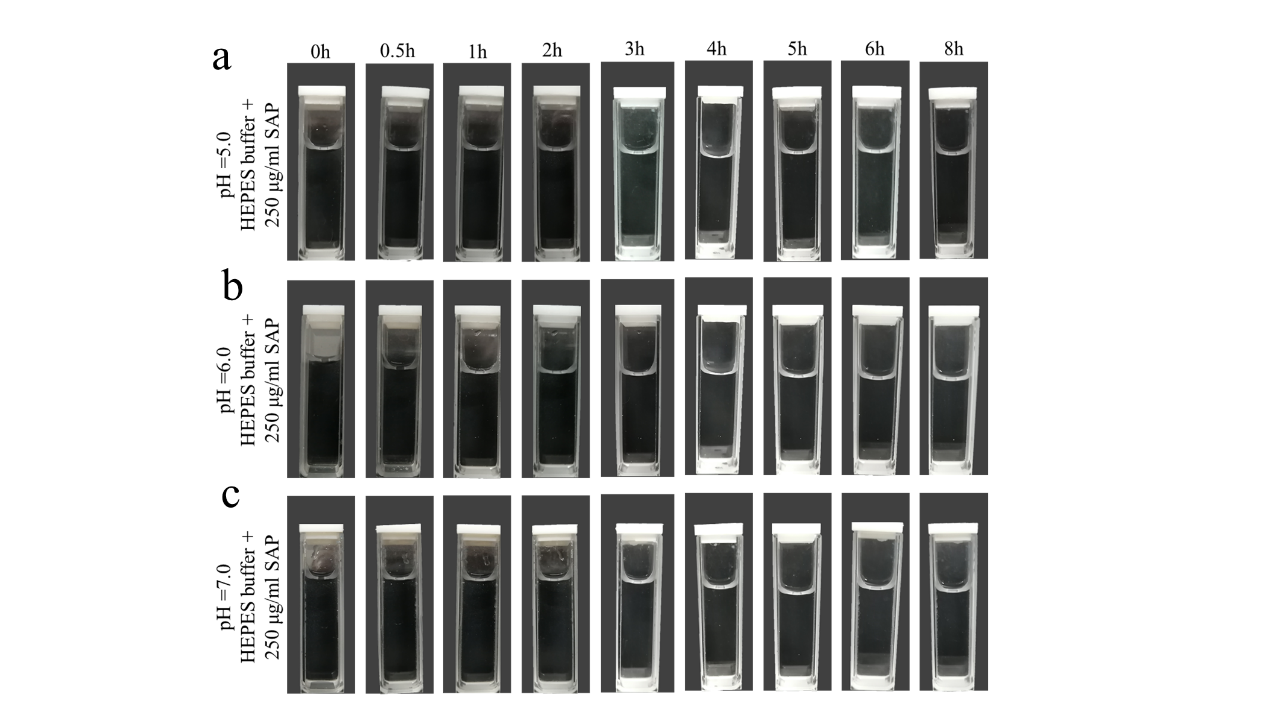


**Figure S6** Agglutination assay without bacteria as a control. SAP (250 μg/mL) was added to HEPES buffer at pH=5.0 (a), 6.0 (b) and 7.0 (c).


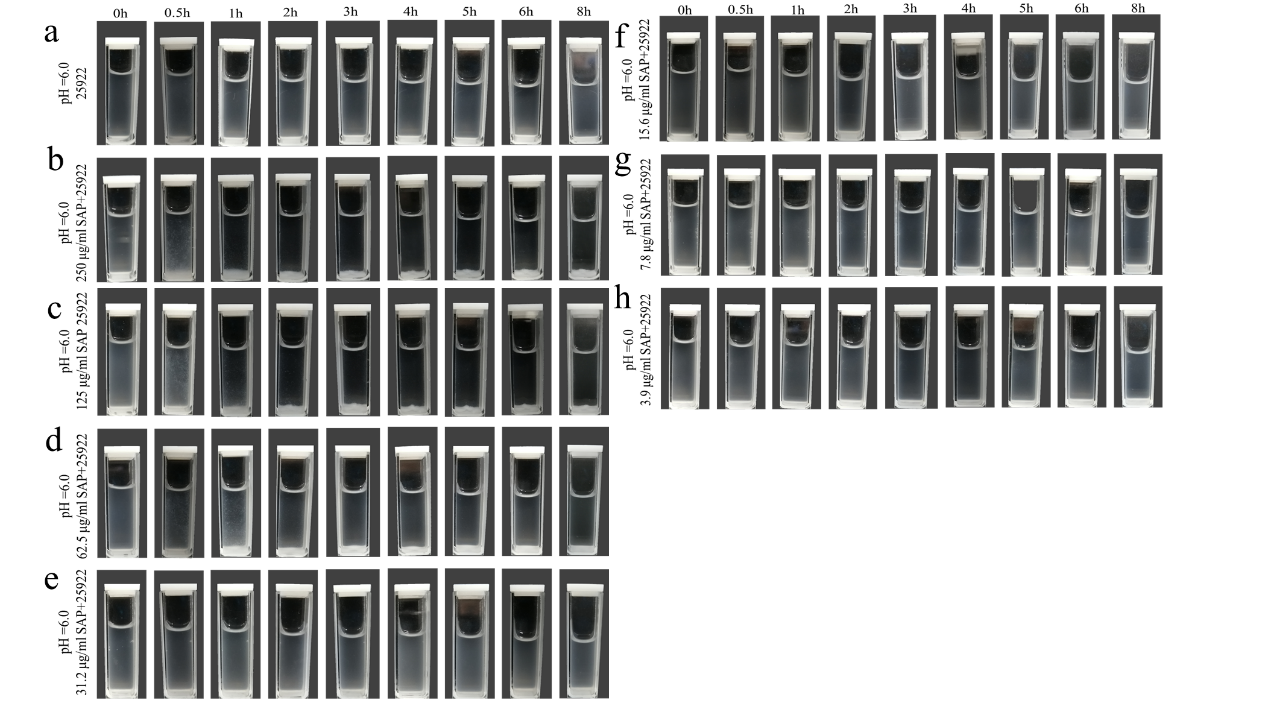


**Figure S7** Bacterial agglutination assay for different concentrations of SAP. Agglutination of *E. coli* ATCC 25922 treated with 0 μg/mL (a), 250 μg/mL (b), 125 μg/mL (c), 62.5 μg/mL (d), 31.5 μg/mL (e), 15.6 μg/mL (f), 7.8 μg/mL (g) and 3.9 μg/mL (h) SAP for 8 h in HEPES buffer at pH=6.0.
